# Comparison of Three Solid Phase Materials for the Extraction of Carboxylic Acids from River Water Followed by 2D GC × GC-TOFMS Determination

**DOI:** 10.1155/2016/6396938

**Published:** 2016-05-04

**Authors:** G. O. Bosire, J. C. Ngila, H. Parshotam

**Affiliations:** ^1^Department of Applied Chemistry, University of Johannesburg, P.O. Box 17011, Doornfontein, Johannesburg 2028, South Africa; ^2^Sustainability Department, Applied Chemistry and Microbiology, Eskom, Private Bag X40175, Cleveland 2022, South Africa

## Abstract

The extraction and determination of aliphatic and aromatic carboxylic acids as well as their influence on the aromaticity and molecularity relationship of natural organic matter (NOM) in water are reported in this study. Three solid phase extraction (SPE) sorbents were used and their extraction efficiencies evaluated after chromatographic determinations (using gas chromatography with a time of flight mass spectrometer (GC × GC-TOFMS) and liquid chromatography with organic carbon detector (LC-OCD)). More than 42 carboxylic acids were identified in raw water from the Vaal River, which feeds the Lethabo Power Generation Station, South Africa, with cooling water. The aromatic carboxylic acid efficiency (28%) was achieved by using Strata*™* X SPE while the highest aliphatic carboxylic acid efficiency (92.08%) was achieved by silica SPE. The hydrophobic nature of NOM in water depends on the nature of organic compounds in water, whether aromatic or aliphatic. The LC-OCD was used to assess the hydrophobicity levels of NOM as a function of these carboxylic acids in cooling water. The LC-OCD results showed that the aromatic nature of NOM in SPE filtered water followed the order Silica>Strata X>C-18. From the results, the hydrophobicity degree of the samples depended on the type and number of carboxylic acids that were removed by the SPE cartridges.

## 1. Introduction

Given the complexity of natural organic matter (NOM) in environmental matrices [1–3], it is has been difficult, over years, to determine its characteristic behaviour in water systems. Studies in the last decade, however, have shown that NOM is made up of fractions that include biopolymers, humic substances, building blocks, low molecular weight neutrals, and low molecular weight acids [4]. This information is of particular importance because of specific chemical moieties or NOM fractions have been shown to have different interactions with other components in water, for example. The low molecular weight acids are of particular interest because they influence water chemistry (pH, ionic strength, and divalent cations), dipole-dipole interactions hydrogen bonding, and complexation reactions [5]. Undoubtedly, these considerations would make the extraction of these acids challenging. In order to choose the most appropriate extraction method that will be desirably recover most aliphatic and aromatic carboxylic acids from water, this research provided essential preliminary assessment on selected solid phase extraction methods.

Various approaches have been explored to extract carboxylic acids in natural water [6]. Clearly, the determination of carboxylic acids in water calls for an effective primary extractive stage before chromatographic analysis [7]. Some of the extraction approaches reported in literature include precipitation [8], conventional electro-dialysis [9], the liquid-liquid extraction and solid phase extraction (SPE) [10–12]. Despite all the advantages of the above mentioned methods, they still suffer from shortcomings. For example, the liquid-liquid separation techniques use solvents whose distribution coefficient are low [13, 14]. A number of SPE sorbents that extract carboxylic acids in water are commercially available [15, 16]. However, the practical merits of various extraction methods have not been exhaustive. The liquid chromatography with organic carbon detection (LC-OCD), in this study, provided information on the changes in hydrophobicity/aromaticity of NOM after the SPE separation. This would in turn confirm the removal of the carboxylic acids from water.

In this present work, we aimed to assess the extraction efficiencies of Silica, C-18 and Strata X resins for recovery of aromatic and aliphatic acids in raw water used for cooling purposes in typical power generating stations in South Africa. In addition, the aromatic/hydrophobic characteristics of the water before and after SPE extraction were compared.

Clearly, the determination of carboxylic acids in water calls for an effective primary extractive stage before chromatographic analysis [18]. Some of the extraction approaches reported in literature include precipitation [19], conventional electrodialysis [20], the liquid-liquid extraction, and solid phase extraction (SPE) [6–8]. Despite all the advantages of the above-mentioned methods, they still suffer from shortcomings. For example, the liquid-liquid separation techniques use solvents whose distribution coefficients are low [9, 10]. A number of SPE sorbents that extract carboxylic acids in water are commercially available [11, 12]. Few studies, however, report on extraction of carboxylic acids followed by chromatographic determinations. The liquid chromatography with organic carbon detection (LC-OCD), in this study, provided information on the changes in hydrophobicity/aromaticity of NOM after the SPE separation. This would in turn authenticate the removal of the carboxylic acids from water.

This study sought to assess the extraction efficiencies of silica, C-18, and Strata X resins for recovery of aromatic and aliphatic acids in raw water used for cooling purposes at Eskom, South Africa. In addition, the aromatic/hydrophobic characteristics of the water before and after SPE extraction were compared. This is part of a larger study that seeks to characterize NOM in cooling water, a step forward in building up databases of significant NOM fractions (including carboxylic acids), which govern chemical reactions that control scale formation in pipes. Eventually, an effective modelling strategy could be developed to monitor these reactions in water, at different physicochemical conditions with the view of scale minimization [4, 13]. The latter was used to assess the hydrophobicity/hydrophilicity levels of NOM as a function of these carboxylic acids in industrial cooling water.

## 2. Experimental

### 2.1. Reagents and Sorbents

In the present study, silica based C-18, silica, and Strata X resins were employed in the extraction of aliphatic and aromatic carboxylic acids prior to their GC × GC-TOFMS determination and LC-OCD assessment. All organic solvents were of GC-MS grade. Some of these (acetone, methylene chloride, tetrahydrofuran, and methanol) were obtained from Merck (Darmstadt, Germany), while others (n-hexane and n-propanol) were obtained from Sigma Aldrich (Germany). The Strata X and C-18 SPE resins were obtained from Phenomenex (Torrance, CA 90501-1430, USA). The synthesis of these SPE sorbents is reported in literature [14, 15].

### 2.2. Solid Phase Extraction

Three critical steps were followed in each extraction: (a) conditioning of the sorbent; (b) sample loading and isolation of the analytes; and (c) the elution step [14]. The cartridges were conditioned with 5 mL methanol and then followed by 5 mL of deionised water. Approximately 1.5-litre raw water samples were separately loaded onto the SPE columns at a rate of 10 mL/min using the vacuum manifold apparatus. The analytes were eluted from Strata X SPE by 5 mL acetone, followed by 5 mL of methylene chloride, and from the silica based cartridges using a mixture of tetrahydrofuran (THF) and methanol in a 1 : 1, v/v ratio. The eluted samples were dried in a controlled lamina flow apparatus at 25°C, reconstituted using pure methylene chloride (GC-grade), and thereafter injected into the GC for determination.

### 2.3. GC × GC-TOFMS

The determination of carboxylic acids from water after SPE extracted was performed using GC × GC gas chromatograph (Pegasus 4D, LECO Corporation, South Africa) with a time of flight mass spectrometer (TOFMS). The GC technique employed the RXi 5Sil-MS primary column (40 m long with internal diameter (ID) of 0.25 mm and 0.25 *μ*m film thickness) and the RXi 17Sil-MS secondary dimension column (4 m long with an ID of 0.25 mm and the film thickness of 0.25 *μ*m). Nitrogen gas, compressed air, and liquid nitrogen were used for quad-jet thermal modulator operation. The injector temperature for samples was set at 280°C, at a volume of 1 *μ*L on a splitless mode. Helium was used as the carrier gas at a flow rate of 1.0 mL/min and a head pressure of 90 kPa. The modulator interface temperature was set at 30°C above the secondary oven temperature. The transfer line temperature was set at 300°C and the ion source temperature at 240°C. Electron impact ionization energy was set at −70 eV with offset of 300 V totaling to the detector voltage of 1,600 V. The first-dimension column temperature was started at 55°C and held for 4.5 min, then increased to 280°C at a ramping rate of 10°C/min, and held for 4.5 min, whereas the secondary column temperature started at 75°C held for 4.5 min, then ramped to 310°C at a ramping rate of 10°C/min, and held for 4.5 min. The data was processed by identifying aliphatic and aromatic carboxylic acids with their respective unique compound masses (Q masses) on spectral databases and first- and second-dimension retention times. The MS analysis was carried out at mass range of 40–500 amu and acquisition rate was 100 spectra/sec.

### 2.4. The Liquid Chromatography-Organic Carbon Detection (LC-OCD)

Raw water samples filtered through SPE cartridges were analysed using LC-OCD (DOC-Labor, Karlsruhe, Germany). The raw water samples were obtained from the Vaal River which feeds Lethabo Power Station with cooling water. The LC-OCD technique, developed to identify fractions natural organic matter in water, gives quantitative information and qualitative results regarding molecular size distribution of organic matter in water [17]. The technique separates components on the basis of their molecular size [4]. Water samples were injected into a column filled with a chromatographic gel material where large molecules were eluted first followed by the smaller compounds [17]. To achieve quantification of organic carbon, organic nitrogen, and specific UV absorbance at *λ*-254, their responses in the samples at different retention times were measured with an organic carbon detector, organic nitrogen detector, and UV detector, respectively.  Table 1 shows fractions of the natural organic matter components based on their retention times and peak areas [4]. After inline filtration through 0.45 *μ*m, the LC-OCD analysis was carried out following the procedure described by Huber et al., 2011 [4].

## 3. Results and Discussion

Results obtained from samples extracted by silica, C-18, and Strata X cartridges and determined by first- and second-dimension chromatographic separation are presented in this section. The unique masses (Q masses), retention times, library hit similarities, and peak areas for selected carboxylic acids (especially aromatic acids) are shown in Table 2.

**Figure 1:**
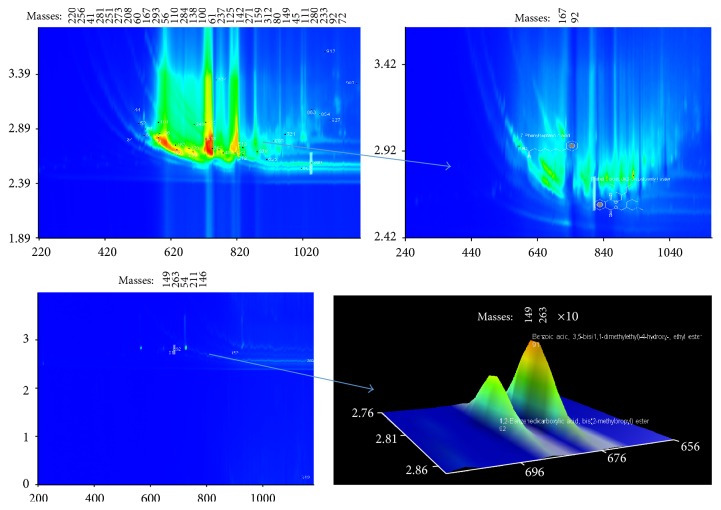
2D chromatograms showing the compounds extracted in raw water using silica SPE and determined by using deconvolution ChromaTOF software in the GC × GC-TOFMS technique. Aromatic acids are shown in zoomed chromatograms to the right and indicated by arrows. The respective names, retention times, and GC-library similarities of selected carboxylic acids are tabulated (Table 2).

**Figure 2:**
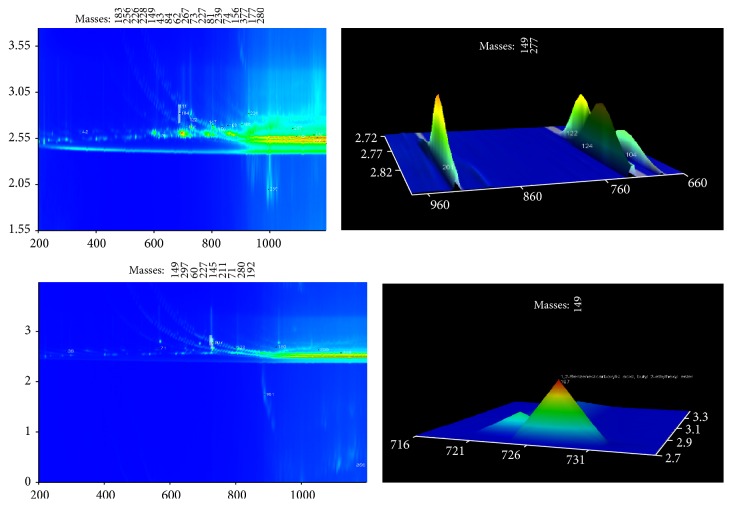
2D chromatograms showing the compounds extracted in raw water using C-18 SPE and determined by using deconvolution ChromaTOF software in the GC × GC-TOFMS technique. Aromatic acids are shown as zoomed chromatograms to the right and indicated by arrows. The respective names, retention times, and GC-library similarities of selected compounds are tabulated (Table 2).

**Figure 3:**
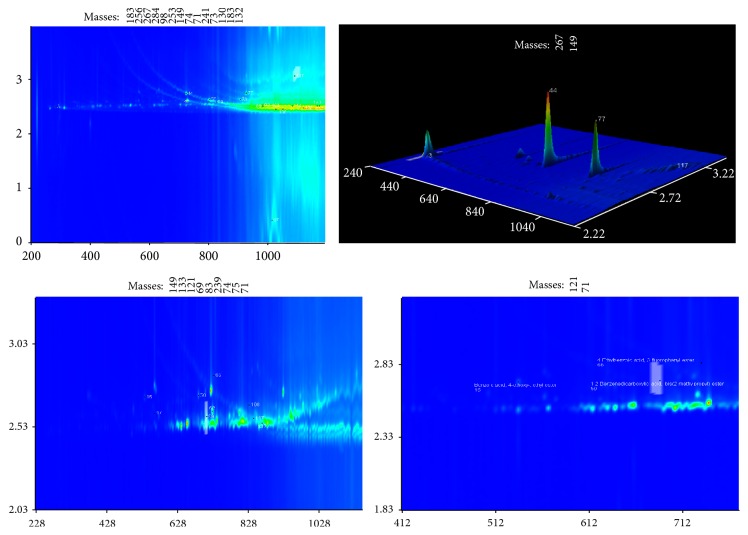
2D chromatograms showing the compounds extracted in raw water using silica SPE and determined by using deconvolution ChromaTOF software in the GC × GC-TOFMS technique. Aromatic acids are shown as zoomed chromatograms to the right and indicated by arrows. The respective names, retention times, and GC-library similarities of selected carboxylic acids are tabulated (Table 2).

**Figure 4:**
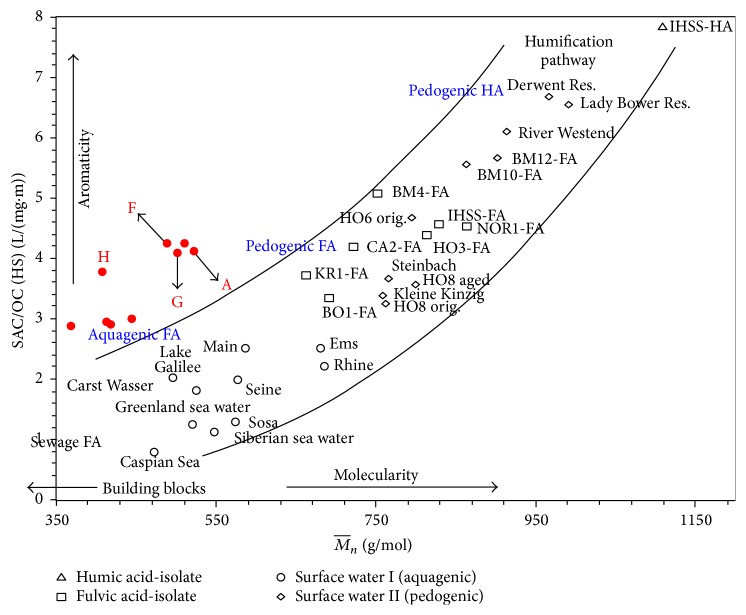
The aromaticity-molecularity relationship presented on a HS diagram. A represents raw water from the Vaal River, F represents raw water filtered through silica, G represents raw water filtered through X SPE, and H represents water filtered through C-18 SPE. HS diagram presented here reveals the aromatic character (*x*-axis) of NOM fractions as a function of their molecular weights (*y*-axis). This diagram plots the spectral absorption coefficient (SAC) to the organic carbon (OC) ratio (aromaticity) of a HS fraction against its nominal molecular weight (*M*_*n*_) [4].

The chromatograms of the total carboxylic acids and aromatic carboxylic acids are provided separately in the same figures (Figures 1, 2, and 3). Using deconvolution ChromaTOF software and the structured unique masses (Q masses) spectra in GC × GC-TOFMS [16, 21, 22], the coeluting carboxylic acids were also identified. Hitherto, a comparison, in terms of aliphatic and aromatic carboxylic acid extraction efficiency, was evaluated and results are discussed in this section. The hydrophilic or hydrophobic changes occurring in water after removal of carboxylic acids are shown in Table 3 and Figure 4.

### 3.1. Silica SPE Extracted Carboxylic Acids

As shown in Table 2, raw water using for cooling at Eskom chiefly contains straight-chain and branched carboxylic acids (all referred to as aliphatic carboxylic acids). The results showed a total of 42 carboxylic acids extracted by silica SPE from the Vaal River water. Of these, there are aliphatic carboxylic acids (90.48%) while aromatic acids were 9.52% of the total (Table 2 and Figure 1). These aromatic acids included 1,2-benzenedicarboxylic acid, bis(2-methyl) propyl ester; benzoic acid, 3,5-bis(1,1dimethylethyl)-4-hydroxy-ethyl ester; phthalic acid, di(2-propylpentyl) ester, and 7-phenylheptanoic acid. Comparatively, the number of aromatic carboxylic acids recovered by the silica SPE cartridge was lower than those recovered by C-18 (25%) and Strata X (28%) cartridges.

A possible explanation for the relatively large number of carboxylic acids in this river could be degradation and aggregation. Related studies have reported short and medium chain carboxylic acids due to anaerobic fermentation of organic matter [16, 18]. Clearly, microbial activity and changing physicochemical parameters (such as pH, ionic strength, and temperature) influence the distribution of NOM fractions. The result of this influence explains the fact that higher molecular weight and aromatic compounds in water degrade and aggregate forming smaller intermediates, which include carboxylic acids of varying molecular weights.

Figure 1 also shows the coeluting and closely eluting compounds. They include eicosanoic acid (1st *T*_*R*_ = 728; 2nd *T*_*R*_ = 2.800); hexanedioic acid, bis(2-ethylhexyl) ester (1st *T*_*R*_ = 728; 2nd *T*_*R*_ = 2.950), eicosanoic acid, 2,3-bis(acetyloxy)propyl ester (1st *T*_*R*_ = 840; 2nd *T*_*R*_ = 2.650), 9,12,15-octadecatrienoic acid, 2-(acetyloxy)-1-[(acetyloxy)methyl]ethyl ester, (1st *T*_*R*_ = 840; 2nd *T*_*R*_ = 2.800); octanedioic acid (1st *T*_*R*_ = 1048; 2nd *T*_*R*_ = 2.800), decanoic acid, 2-propenyl ester (1st *T*_*R*_ = 1048; 2nd *T*_*R*_ = 3.070). Some other carboxylic acids were eluted at different times in the primary dimension but at the same time in the secondary dimension. These included benzoic acid, 3,5-bis(1,1-dimethylethyl)-4-hydroxy-ethyl ester (1st *T*_*R*_ = 676; 2nd *T*_*R*_ = 2.800); octanedioic acid (1st *T*_*R*_ = 1048; 2nd *T*_*R*_ = 2.800) and 1,2-benzenedicarboxylic acid, bis(2-methyl) propyl ester (1st *T*_*R*_ = 688; 2nd *T*_*R*_ = 2.810).

### 3.2. C-18 SPE Extracted Carboxylic Acids

The total carboxylic acids extracted by the C-18 resin were 20, 15 of which were aliphatic and 5 aromatic (Table 2 and Figure 2). The aromatic acids included 1,2-benzenedicarboxylic acid, bis(2-methylpropyl) ester (1st *T*_*R*_ = 688; 2nd *T*_*R*_ = 2.820); benzenepropanoic acid, 3,5-bis(1,1-dimethylethyl)-4-hydroxy-, methyl ester (1st *T*_*R*_ = 1080; 2nd *T*_*R*_ = 2.660); phthalic acid, di(oct-3-yl) ester (1st *T*_*R*_ = 1136; 2nd *T*_*R*_ = 2.570); 1,2-benzenedicarboxylic acid, butyl 2-ethylhexyl ester (1st *T*_*R*_ = 1152; 2nd *T*_*R*_ = 2.590); phthalic acid, butyl undecyl ester (1st *T*_*R*_ = 1188; 2nd *T*_*R*_ = 2.520). Of these aromatic acids, only 1,2-benzenedicarboxylic acid, bis(2-methylpropyl) ester was extracted by both silica and C-18 solid phase extraction methods.

Aliphatic carboxylic acids were less retained on C-18 (75%) compared to silica SPE (90.48%). This is because C-18 consists of a silica surface functionalized with hydrophobic groups, which favour retention of the carboxylic acids in the reversed-phase [23, 24]. Additional studies have reported the application of the C-18 solid-phase in extraction of organic acids using the reverse phase and henceforth higher retention of those with higher hydrophobicities. The retention efficiency of C-18 was 25% for the aromatic carboxylic acids (relatively more hydrophobic) compared to silica's 9.52%. The remaining 75% of the carboxylic acids extracted using C-18 were mainly medium and long chain aliphatic acids. Except for cis-13-octadecenoic acid (1st *T*_*R*_ = 712; 2nd *T*_*R*_ = 2.670) and 9,12-octadecadienoic acid (1st *T*_*R*_ = 716; 2nd 20 *T*_*R*_ = 2.750) that had similar retention times, the time of flight rapid separation and mass spectral signatures did not show other closely eluting or coeluting compounds.

### 3.3. Strata X Extraction

This Strata X polymeric resin has the capacity to adsorb both polar and nonpolar compounds with more capacity for the former [25]. It consists of copolymers that have aromatic rings in their structures which allow for electron-donor interaction between the sorbent and the *π* bonds in the analyte. Therefore, this sorbent overcomes many limitations of bonded silicas because their hydrophobic surface contains a relatively large number of active aromatic sites that allow these *π*-*π* interactions with aromatic carboxylic compounds. The results obtained in this study are in good agreement with literature. More aromatic carboxylic acids were retained in the Strata X than the two other sorbents (Table 2 and Figure 3). Except for m-hydroxymandelic acid, tris(trimethylsilyl), all the other aromatic acids extracted by Strata X had >70% GC-library hit similarity. The retention efficiency of aromatic acids for Strata X 28% is higher than the one obtained using C-18 (25%) and silica (9.52%). Table 2 shows a total of 25 carboxylic acids extracted by Strata X with 18 aliphatic carboxylic acids and 7 aromatic carboxylic acids. The Strata X extracted 72% aliphatic acids of the total carboxylic acids and 28% aromatic acids. Two coeluting aliphatic carboxylic acids were separated in the second dimension. These were cis-10-heptadecenoic acid (1st *T*_*R*_ = 984; 2nd *T*_*R*_ = 2.520) and E-9-tetradecenoic acid (1st *T*_*R*_ = 984; 2nd *T*_*R*_ = 2.540) (Figure 3).

### 3.4. LC-OCD Characterization

First, the LC-OCD analyses were performed to identify the major fractions of NOM in raw water. Second, results showed that the concentration of NOM fractions decreased after filtration through the three SPE methods, that is, silica, C-18, and Strata X (Table 2). The decrease was more pronounced in C-18 filtered water and followed the order C-18>silica>Strata X.

The relative degrees of aromaticity against molecularities of NOM in the four water samples are presented in the humic substances diagram (HS diagram). From the results, the hydrophobicity degree of the samples depended on how much hydrophilic carboxylic acids were removed. It is worth noting that molecules with aromatic rings have higher hydrophobicities. Figure 4, which is the humic substances diagram (HS diagram), shows the relationship between molecularity and aromaticity of organic compounds in the water samples. The concentrations of the hydrophobic component followed the order Strata X>C-18>silica>raw water and the hydrophilic fraction followed the order raw water>silica>Strata X >C-18. From Figure 4, more aromatic compounds were extracted by C-18 (H on the HS diagram) followed by Strata X (F on the HS diagram). However the molecular weights of NOM components remaining in the water were higher in water extracted by Strata X. This agrees with data obtained using 2-dimensional GC.

Hydrophobicity of samples was also assessed using the specific UV absorbance at the wavelength of 254 (SUVA). The various SUVA values and the composition of the samples are tabulated in Table 3. Natural organic matter in water is reported to possess a hydrophobic characteristic, when the measured SUVA values are >4 [26, 27]. Accordingly, the aromaticity of the water samples followed the order F>G>H and the molecularity values followed the order G>F>H.

## 4. Conclusions

Effective characterization of raw water samples was achieved by SPE prior to GC × GC-TOFMS determination. The use of Strata-X cartridge resulted in the best recovery of aromatic carboxylic acids from raw water. This was relatively higher recovery and could be attributed to the fact that Strata X is a polymeric resin with aromatic rings in its hydrophobic structure. The silica SPE cartridge was the best in the recovery of aliphatic carboxylic acids. The total number of carboxylic acids extracted by silica SPE was 42, with aromatic acids comprising 9.52% of the total and the rest being aromatic. Accurate determination of the various carboxylic acids was achieved by the first- and second-dimension gas chromatography. The LC-OCD results confirmed that the aromaticity degrees and molecular weights NOM changed after extraction of carboxylic acids. Largely, the hydrophobicity degree of the samples depended on the type and number of carboxylic acids that were removed by the SPE cartridges.

## Figures and Tables

**Table 1:** Characteristic NOM fractions of surface water described by the LC-OCD technique [4, 17].

Fraction	Molecular mass	Characteristics
Biopolymers	>50,000–2,000,000 g/mol	Mostly polysaccharides; not UV-absorbing; may be associated with amino acids and protein
Humic substances	100–10,000 g/mol	Consisting of humic acids (nonsoluble in acids, soluble in basis) and fulvic acids (soluble in acids and basis) in varying amounts
Building blocks	350–500 g/mol	Intermediates in degradation process fulvic acids
Low molecular weight organic acids (LMWAs)	>1 *μ*m	Final degradation products of organics, but also released by algae and bacteria
Low molecular mass neutrals and (LMWN) amphilics	>1 *μ*m	Slightly hydrophobic substances, such as alcohols, aldehydes, ketones, and amino acids
Hydrophobic compounds (HOC)	>1 *μ*m	Difference between TOC and summarised chromatographic fractions; not detectable by chromatography; consisting probably of natural lipids, lipoids, and hopanoids

**Table 2:** Selected carboxylic acids extracted by silica, C-18, and Strata X.

GC × GC-TOFMS determinations						
Peak #	Name	Unique mass	Weight	Retention times (1st, 2nd) (sec)	Library similarity (%)	Area
	Silica SPE					
91	Benzoic acid, 3,5-bis(1,1dimethylethyl)-4-hydroxy-ethyl ester	263	278	676, 2.8	75.2	133966
92	1,2-Benzenedicarboxylic acid, bis(2-methyl) propyl ester	149	278	688, 2.81	93.1	406649
106	Cyclobutanecarboxylic acid, 2-tridecyl ester	80	226	588, 2.850	70.5	495527
152	Phthalic acid, di(2-propylpentyl) ester	146	370	884, 2.730	75.0	7249
731	7-Phenylheptanoic acid	233	436	968, 2.840	79.9	117828

	C-18					
104	1,2-Benzenedicarboxylic acid, bis(2-methylpropyl) ester	149	278	688, 2.820	93.4	313930
287	Benzenepropanoic acid, 3,5-bis(1,1-dimethylethyl)-4-hydroxy-, methyl ester	156	254	1080, 2.660	76.1	9054.8
316	Phthalic acid, di(oct-3-yl) ester	183	282	1136, 2.570	84.6	90552
330	1,2-Benzenedicarboxylic acid, butyl 2-ethylhexyl ester	280	280	1152, 2.590	74.7	4870.7
350	Phthalic acid, butyl undecyl ester	256	256	1188, 2.520	89.9	517199

	Strata X					
15	Benzoic acid, 4-ethoxy-, ethyl ester	121	194	536, 2.710	88.5	126885
117	1,2-Benzenedicarboxylic acid, bis(8-methylnonyl) ester	149	446	1092, 3.08	74.9	310
44	Benzenedicarboxylic acid, butyl 2-ethylhexyl ester	149	334	724, 3.70	86.8	1045431
77	Phthalic acid, di(oct-3-yl) ester	149	390	928, 2.76	89.3	643832
66	4-Ethylbenzoic acid, 3-fluorophenyl ester	133	244	732, 2.840	85.3	126054

**Table 3:** Raw and SPE filtered water characteristics.

Sample	SUVA = SAC/DOC (L/(mg*∗*m))	DOC (ppb)	Fractions of dissolved organic carbon (DOC) (in %)			
			Hydrophobic DOC	Hydrophilic DOC	Building blocks	Low molecular weight acids (LMWAs)
Raw water	3.40	9106	7.3	92.7	12.2	NQ
Silica filtered water	2.88	1892	14.5	85.5	8.5	0.1
C-18 filtered water	1.37	6339	14.7	77	4.7	NQ
Strata X filtered water	2.93	1942	16.3	83.7	8.8	9.7

SUVA: specific ultraviolet absorbance.

SAC: spectral absorption coefficient.

DOC: dissolved organic carbon.
